# Cyclic drying and wetting tests on combined remediation of chromium-contaminated soil by calcium polysulfide, synthetic zeolite and cement

**DOI:** 10.1038/s41598-021-91282-4

**Published:** 2021-06-02

**Authors:** Xilin Li, Xiaowan Yu, Ling Liu, Jianlin Yang

**Affiliations:** 1 School of Civil Engineering, Liaoning Technical University, Fuxin, 123000 Liaoning Province China; 2 College of Materials Science and Engineering, Liaoning Technical University, Fuxin, 123000 Liaoning Province China

**Keywords:** Ecology, Environmental sciences, Materials science

## Abstract

Using calcium polysulfide as the reducing agent, synthetic zeolite as the adsorbent, and cement as the curing agent, the dual-index orthogonal test method was used to determine the best remediation dosage of chromium-contaminated soil. On this basis, through the dry–wet cycle test, the durability of the chromium-contaminated soil after repair is analyzed from the perspectives of unconfined compressive strength, toxic leaching concentration, quality loss, and microscopic characterization. Test results showed that the optimal ratio for the joint repair of chromium-contaminated soil was 3 times the amount of CaS_5_, 15% synthetic zeolite, and 20% cement. With the increase in the number of wet–dry cycles, the unconfined compressive strength of the composite preparation combined to repair chromium-contaminated soil was first increased and then reduced, and the concentration of Cr(VI) and total chromium in the leachate was first decreased and then increased. The higher the chromium content of the contaminated soil was, the lower the unconfined compressive strength, and the higher the leaching concentration of Cr(VI) and total chromium were. With the increase in cycle times, the cumulative mass-loss rate of composite preparations for repairing chromium-contaminated soil gradually increased, and the higher the chromium content was, the higher the cumulative mass-loss rate, which was less than 2%, reflecting the combination of composite preparations for repairing chromium-contaminated soil to have good durability. Microscopic and macroscopic results are consistent with each other.

## Introduction

Chromium salt is widely used as raw material in many industries, such as the mining, chemical, metallurgy, electroplating, leather, ceramic, dyeing, anticorrosion, and medical industries, and a large amount of highly toxic solid waste chromium residue is discharged in its long-term production process^1,2^. According to statistics, China has produced more than 6 million tons of chromium residue, and every ton of chromium residue produces 2.5–10 tons of chromium-contaminated soil. On the basis of this estimate, China’s soils that are heavily polluted by chromium that amounts to 15–60 million tons, leaving a huge environmental “tumor” to society^3^. The state must take corresponding measures to remedy this, so that it not only meets the environmental requirements for human survival, but also the needs of production and construction, or the contaminated soil after repair meets the requirements for roadbed fillers and construction materials, so as to realize waste-recycling-resource-modified repair mode.

Remediation techniques for heavy-metal chromium-contaminated soil mainly include the solidification/stabilization, chemical-reduction, chemical-cleaning, bioremediation, and electric-remediation methods^4,5^. From current research, a single method cannot effectively repair chromium-contaminated soil. Considering the characteristics of the immobilization/stabilization and chemical-reduction methods, a combination of both first uses a reducing agent to reduce the highly toxic Cr(VI) to lower the toxicity of Cr(III). Then, a curing agent is used for curing/stabilization treatment, which has important guiding significance to solve the problem of chromium-contaminated-soil repair. With regard to reducing-agent selection, calcium polysulfide (CPS) is very stable in alkaline environments, has strong reducing activity, is easy to obtain, and is simple to prepare, making it a reducing agent with strong application prospects^6,7^. In terms of curing-agent selection, ordinary Portland cement (OPC) is a widely used curing agent in heavy-metal-contaminated soil^8,9^. A reducing agent is used to change the high virulent concentrations of Cr(VI). After reduction, toxicity to Cr(III) was much lower, Cr(III) content in soil increased dramatically, and cement was solidified, so high concentrations of Cr(III) also could result in the precipitation of Cr(III). Cr(III), under certain conditions can be converted to Cr(VI), but there is still a potential risk that its long-term repair effect is not ideal^10,11^. Considering the need for the resource utilization of fly ash and the strong adsorption performance of zeolite for Cr(III), the research team used the alkali fusion–hydrothermal method to synthesize zeolite from fly ash in the early stages. In the remediation of high-concentration chromium-contaminated soil, the first use of reducing agent calcium polysulfide reduced the highly toxic Cr(VI) to the less toxic Cr(III), and then used mine solid waste fly ash to synthesize zeolite to adsorb high concentration of Cr(III), and solidify it with the curing-agent cement. Chromium-contaminated soil can be repaired through reduction–adsorption–solidification. After repair, the solidified contaminated soil can be used as civil-engineering material for comprehensive utilization^12^.

In fact, solidified heavy-metal-contaminated soil is often in a complex and changeable natural environment during resource utilization. The solidified soil inevitably suffers from wind, sun, and rain, seasonal freezing and thawing, carbonization, acid-rain invasion, and chemical erosion. Frequent abnormal climate changes (extremely severe cold/heat, concentrated rainstorms) in China in recent years have caused heavy-metal-contaminated soils to be eroded by dry and wet cycles. Especially in northern areas, it is rainy in summer, and dry and windy in spring. The soil swells and shrinks due to water absorption, which reduces the compressive strength of solidified soil. Research on the mechanical stability and chemical stability of solidified soil under alternate dry and wet conditions aims to realize its engineering uses.

A large number of studies showed that dry–wet cycles deteriorate the mechanical properties of solidified soil and increase the risk of heavy-metal leaching. Zha et al.^13^ found that the dry–wet cycle effect reduces the strength characteristics of OPC-solidified soil, and increases the leaching risk of Pb and Zn in the solidified body. Cao et al.^14^ added cement and lime, two curing agents, to lead-contaminated soil and conducted dry–wet cycle tests. Results showed that the dry–wet cycle durability of cement-stabilized soil was slightly better than that of lime-stabilized soil, and soil-moisture content is one of the key parameters to ensure the reinforcement effect. Studies by Yoshimichi et al.^15^ showed that the anion-chain polymerization of silicate in the hardened cement slurry makes the structure of calcium silicate hydrate (CSH) more compact, and the specific surface area is significantly reduced during a dry–wet cycle, especially during drying. Pores increase, and the cement-based material shrinks and cracks. Li et al.^16^ used dry–wet-cycle tests to evaluate the durability of solidified sludge materials with bentonite additives. Studies found that the proper blending of bentonite enhances the dry and wet durability of the solidified body, while the excessive blending of bentonite reduces it. Cement also has the problem of minimal content. Yulin Bo et al.^17^ obtained Ground Granulated Blast Furnace Slag(GGBS) + MgO-modified clay by mixing MgO and GGBS. Meanwhile, the same amount of cement-modified clay was used as a reference sample to study the influence of dry–wet cycle on the unconfined strength of GGBS + MgO-modified clay. With the increase of the number of dry–wet cycles, the strength of GGBS + MgO-modified clay was decreased, the damage of the sample increased, and the pH value was significantly decreased. In contrast, cement-modified clay has relatively better dry and wet resistance. Wang et al.^18^ studied the effect of curing temperature on the leaching behavior and durability of GBS–MgO–CaO (GMC) stable/GMC-cured Pb/Zn-contaminated clay soil. Results showed that increasing the curing temperature, curing time, and the adhesive amount can increase the leaching rate and durability of all samples. Compared with cement curing alone, GMC has better chemical stability. Li et al.^19^ used a dry–wet cycle on the impact of ordinary Portland cement (OPC) treatment of lead-contaminated soil. Results showed that, as the number of dry–wet cycles increased, the cumulative mass loss of the sample increased linearly, while the pH value of the leachate dropped. The leaching and deterioration of the cured samples increased with the increase in acidity. Liu et al.^20^ used low-temperature nitrogen adsorption and desorption, cadmium leaching, and permeability to change the performance of cement-solidified Cd-contaminated soil under a dry–wet cycle, and focused on the occurrence and evolution of cracks.

At present, research on solidified heavy-metal-contaminated soil mostly focuses on its engineering properties, and the stability of solidified soil under a dry–wet cycle is not perfect. The durability of high-concentration highly toxic heavy-metal chromium-contaminated soil under dry–wet cycles has not been reported. There are still many controversies about the microscopic mechanism of changes in the engineering properties of solidified heavy-metal-contaminated soils during a dry–wet cycle, and there is no unified understanding.

In order to explore the effect of dry–wet cycles on the mechanics and leaching characteristics of calcium polysulfide–fly ash synthetic zeolite–cement joint repair of heavy-metal chromium-contaminated soil, this article tests the unconfined compressive strength and leaching characteristics of chromium-contaminated soil in a dry–wet cycle after repairing, combined with microscopic scanning electron microscopy and XRD analysis to study the combined repair of microscopic changes on the compressive strength and leaching characteristics of chromium-contaminated soil after being damaged by wet and dry cycles. Research results provide key theoretical and parameter support for the joint repair of heavy-metal chromium-contaminated soil and resource utilization.

## Results and discussion

### Selection of materials for joint repair of chromium-contaminated soil

Table 1 shows the results of the orthogonal test. Range analysis was performed according to the results of Table 1. The range-analysis results are shown in Table 2.

**Table 1:** Orthogonal design scheme and results.

Serial number	Influencing factors			UCS/MPa	Toxic leaching/(mg/L)	
	A/Times	B/%	C/%		Cr(VI)	Total Cr
1	1 (2 Times)	1 (5%)	1 (10%)	6.91	0.47	4.02
2	1 (2 Times)	2 (10%)	2 (15%)	9.18	0.25	1.79
3	1 (2 Times)	3 (15%)	3 (20%)	12.31	0.16	0.92
4	2 (3 Times)	1 (5%)	2 (15%)	9.08	0.06	1.97
5	2 (3 Times)	2 (10%)	3 (20%)	12.24	0.05	0.91
6	2 (3 Times)	3 (15%)	1 (10%)	7.82	0.08	1.83
7	3 (4 Times)	1 (5%)	3 (20%)	12.02	0.01	1.54
8	3 (4 Times)	2 (10%)	1 (10%)	7.28	0.03	2.32
9	3 (4 Times)	3 (15%)	2 (15%)	9.46	0.02	0.74

**Table 2:** Orthogonal test results range analysis calculation table.

	A/Times	B/%	C/%
**Unconfined compressive strength**			
$\overline{{K_{11} }}$	9.47	9.34	7.34
$\overline{{K_{12} }}$	9.71	9.57	9.24
$\overline{{K_{13} }}$	9.59	9.86	12.19
*R*_1_	0.24	0.52	4.85
**Total chromium leaching concentration**			
$\overline{{K_{21} }}$	2.24	2.51	2.72
$\overline{{K_{22} }}$	1.57	1.67	1.50
$\overline{{K_{23} }}$	1.53	1.16	1.12
*R*_2_	0.71	1.35	1.60
**Chromium hexavalent leaching concentration**			
$\overline{{K_{31} }}$	0.29	0.18	0.19
$\overline{{K_{32} }}$	0.06	0.11	0.11
$\overline{{K_{33} }}$	0.02	0.09	0.07
*R*_3_	0.27	0.09	0.12

Table 2 shows that, from the perspective of unconfined compressive strength, the primary and secondary order of the 28 day strength, factors affecting the combined repair of chromium-contaminated soil were cement content → fly-ash synthetic zeolite content → CaS_5_ content. The best test ratio was: CaS_5_ content 3 times, synthetic zeolite content 15%, and cement content 20%. The unconfined compressive strength of the contaminated soil after remediation increased with the increase in cement content, but the relationship between the content of CaS_5_ and synthetic zeolite, and the unconfined compressive strength of the specimen was not very obvious. From the perspective of toxicity leaching, the primary and secondary order of factors affecting the total chromium leaching concentration of the combined remediation of chromium-contaminated soil were cement content → fly-ash synthetic zeolite content → CaS_5_ content. The primary and secondary order of factors affecting the leaching concentration of Cr(VI) in the combined remediation of contaminated soil were CaS_5_ content → cement content → fly-ash synthetic zeolite content. The best test ratios of the total chromium and Cr(VI) toxicity leaching test were: CaS_5_ content is 4 times, synthetic zeolite content 15%, and cement content 20%. Total chromium and Cr(VI) leaching concentration of the chromium-contaminated soil after joint remediation was negatively correlated with the content of CaS_5_, synthetic zeolite, and cement content. The change of total chromium leaching concentration was most significantly affected by cement content and synthetic zeolite. Second, the change of Cr(VI) leaching concentration was most significantly affected by CaS_5_ content. From the perspective of leaching concentration, when reducing agent CaS_5_, adsorbent synthetic zeolite, and curing agent cement were all at maximum, the leaching effect of total chromium and Cr(VI) was best. However, considering the actual engineering cost and dosage of the preparation should be reduced as much as possible for meeting the requirements. Therefore, comprehensive balance analysis determined the optimal ratio for joint repair of chromium-contaminated soil to be 3 times the dosage of CaS_5_, 15% synthetic zeolite, and cement amount 20%.

### Strength change of combined repair of chromium-contaminated soil under action of dry–wet cycle

**Figure 1:**
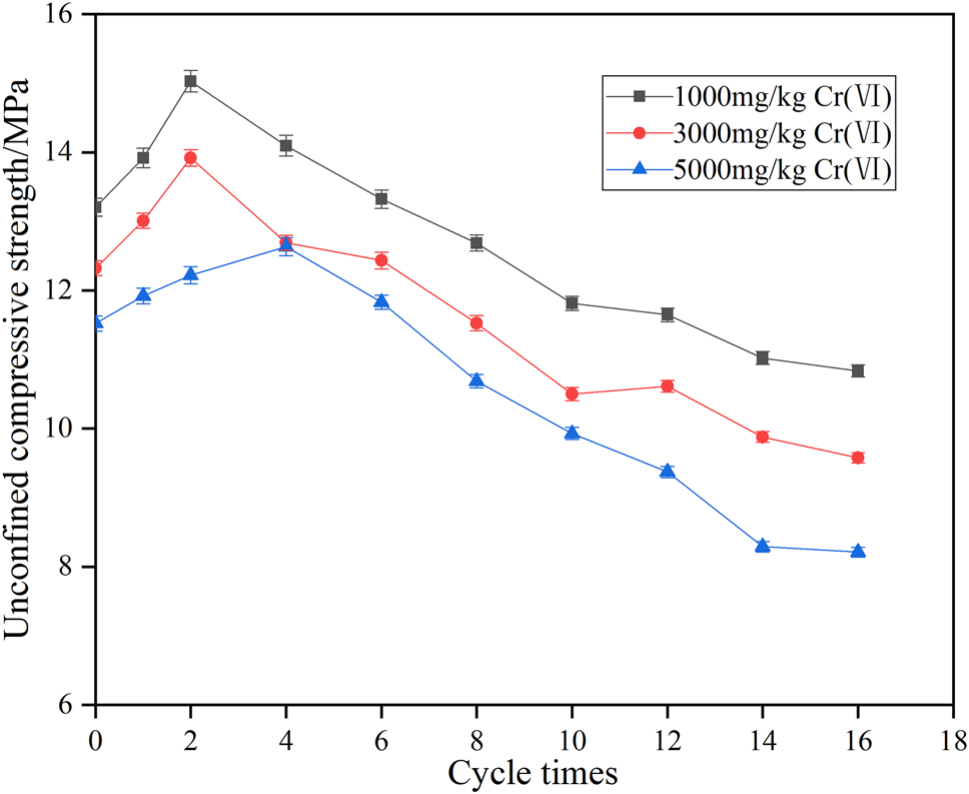
The relationship between unconfined compressive strength and the number of dry wet cycles.

The test compared the variation of unconfined compressive strength with the number of dry and wet cycles under different conditions of chromium content, combined to repair standard specimens of chromium-contaminated soil, and test results are shown in Fig. 1.

Figure 1 shows that, in the beginning, the unconfined compressive strength of the combined repair of chromium-contaminated soil increased with the increase in the number of wet and dry cycles. After reaching the maximal value, it gradually decreased as the number of dry–wet cycles continued to increase. In the initial stage of the dry–wet cycle, the unconfined compressive strength of the combined repair of chromium-contaminated soil increased to varying degrees. For 1000 and 3000 mg/kg of chromium-contaminated soil, the peak of the unconfined compressive strength appeared at 2 times during the dry–wet cycle, and the peak of the unconfined compressive strength of 5000 mg/kg chromium-contaminated soil appeared at 4 dry–wet cycles. After that, unconfined compressive strength gradually decreased with the progress of dry–wet cycles, and the decrease rate became slower. From strength-loss analysis, the higher the chromium content was, the greater the change in strength loss. After 16 wet and dry cycles, the strength-loss rates of 1000, 3000, and 5000 mg/kg chromium-contaminated soil were 17.95%, 22.27%, and 28.73%, respectively, and strength loss was within 30%, showing better water stability^21,22^.

From analysis of the strength-change process, after 28 days of curing for the joint repair of chromium-contaminated soil, the physical and chemical interaction between cement hydrate and soil in the repair preparation was still occurring, as was the strength increase and dry–wet cycle caused by its hydration products. The weakening effect on strength is a dynamic equilibrium process of mutual decline and growth, and the equilibrium state of the two reaction degrees directly affected the strength of solidified chromium-contaminated soil^23^. In the initial stage of the dry–wet cycle, the strength increase caused by the interaction between remediation agent and chromium-contaminated soil continued. At that time, the destructive effect of the dry–wet cycle on the joint repair of chromium-contaminated soil was not significant in comparison. As the number of dry–wet cycles increased, hydration products formed and became stable. Dry shrinkage and wet expansion cause internal stress in the joint repair of chromium-contaminated soil, and the soil has cracks due to internal stress changes. A dry–wet cycle has a relatively destructive effect that is gradually noticeable and resulting in a decrease in strength. After many instances of drying and wetting, the strength of repairing chromium-contaminated soil was decreased and stabilized.

Figure 1 also shows that, compared with low-content chromium-contaminated soil, the high-content chromium-contaminated-soil solidified body strength peak appeared later, and the peak value was low. This is because the higher the chromium ion content was, the more serious the delay of the hydration reaction of the repair agent was, and the more obvious the weakening effect on the strength of the cured body was, which is not conducive to strength growth. The weakening effect of the dry–wet cycle on strength continued to exist, which led to the repaired contaminated soil with a high content of chromium having lower strength.

### Toxic-leaching changes of combined remediation of chromium-contaminated soil under dry–wet cycle

**Figure 2:**
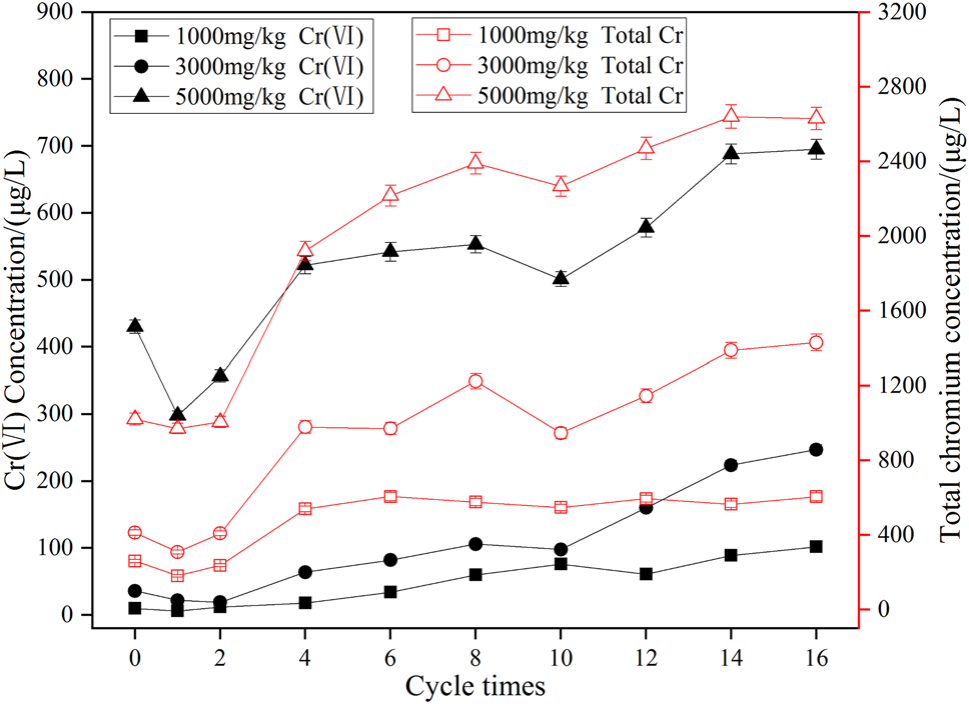
Effect of drying–wetting cycle timeson leaching concentration of Cr.

The experiment compared the variation of hexavalent chromium and total chromium leaching concentration with the number of dry–wet cycles in standard specimens of the combined repair of chromium-contaminated soil under different chromium-content conditions of the contaminated soil. Test results are shown in Fig. 2.

Figure 2 shows that the leaching concentration of Cr(VI) and total chromium decreased in the initial stage of the dry–wet cycle of the remediation of chromium-contaminated soil. After that, as the number of dry–wet cycles increased, leaching concentration also increased, but the content was low (1000 mg/kg). The medium content (3000 mg/kg) of chromium-contaminated soil Cr(VI) and total chromium leaching concentration fluctuated slightly, and the change was relatively stable, while the high content of chromium-contaminated soil (5000 mg/kg) Cr(VI) leaching the concentration fluctuated greatly, and total chromium increased significantly. Compared with the low-content chromium-contaminated soil, the leaching concentration of the solidified body of high-content chromium-contaminated soil was higher.

In the beginning of the dry–wet cycle, the physical and chemical interaction between the cement hydrate and the soil in the repair preparation was still happening. The fly-ash synthetic zeolite had the adsorption effect of metal chromium ions and hydroxide precipitation in the alkaline environment. The formation of chromium ions could meet the requirements of curing/stabilizing chromium ions, and heavy-metal chromium ions are not easy to leach. With the increase in the number of dry–wet cycles, a series of evolutionary processes occurred, such as the expansion of local microcracks, the increase in macropores, the appearance of internal cracks in the contaminated soil, and the appearance of cracks and peeling phenomena on the outside of the contaminated-soil damage. At this time, the contact area between the heavy-metal ions in the contaminated soil and the external environment, especially water, increased, which reduced the ability of the repair agent to adsorb and wrap chromium ions, so that chromium ions were easily leached. In the leaching test, the use of the acidic leaching solution also destroyed the pH balance of the repaired chromium-contaminated soil, the hydrated gel was dissolved and desorbed, and the heavy metals changed, thereby accelerating the leaching of heavy-metal ions^24^.

From analysis of the leaching law shown by the contaminated soil with different chromium content levels, when chromium content in the contaminated soil was low, the remediation agent could effectively solidify/stabilize most of the chromium ions in the soil Cr(VI) and low total chromium leaching. When the chromium content in the contaminated soil was high, the limited content of the repair agent showed an insufficient solidification/stabilization effect of the heavy-metal chromium ions. Because a higher concentration of chromium ions hindered the formation of hydration products of the repair agent, it weakened the adsorption and binding capacity of the hydrated gel. The heavy-metal chromium ions existed in the pores of the contaminated soil in a free state, making the repair agent solidify the chromium ions, the stabilization effect decreased, and the leaching of Cr(VI) and total chromium increased.

Overall, the effect of the dry–wet cycle on the joint repair of chromium-contaminated soil was limited, and the joint repair of chromium-contaminated soil had strong resistance to dry–wet cycles, especially the low- and medium-content chromium-polluted soil.

### Combined repair of quality loss of chromium-contaminated soil under action of dry–wet cycles

**Figure 3:**
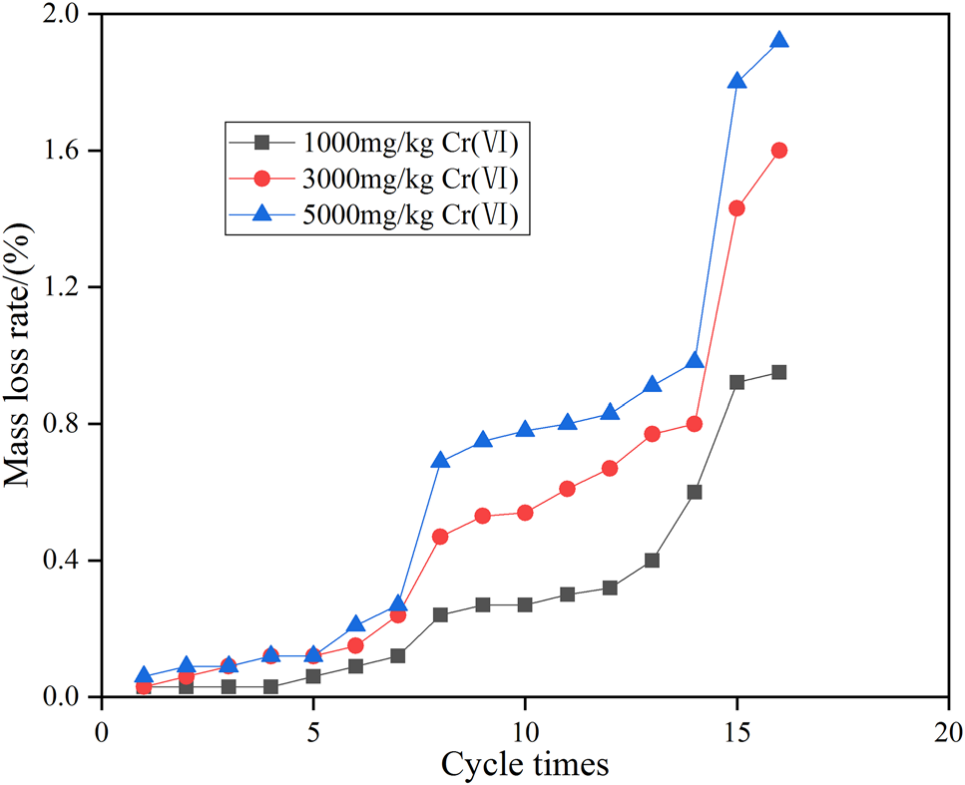
Change of cumulative mass loss rate during dry wet cycle.

The cumulative mass-loss rate of the sample was calculated from Formula (1), and the result is shown in Fig. 3. With the increase in the number of wet and dry cycles, the cumulative mass-loss rate of the composite preparation to repair chromium-contaminated soil gradually increased; and the higher the chromium content of the contaminated soil was, the greater the cumulative mass-loss rate was. The cumulative mass-loss rate of 16 wet and dry cycles was less than 1%, which shows that the joint repair of chromium-contaminated soil had strong resistance to dry and wet cycles.

**Figure 4:**
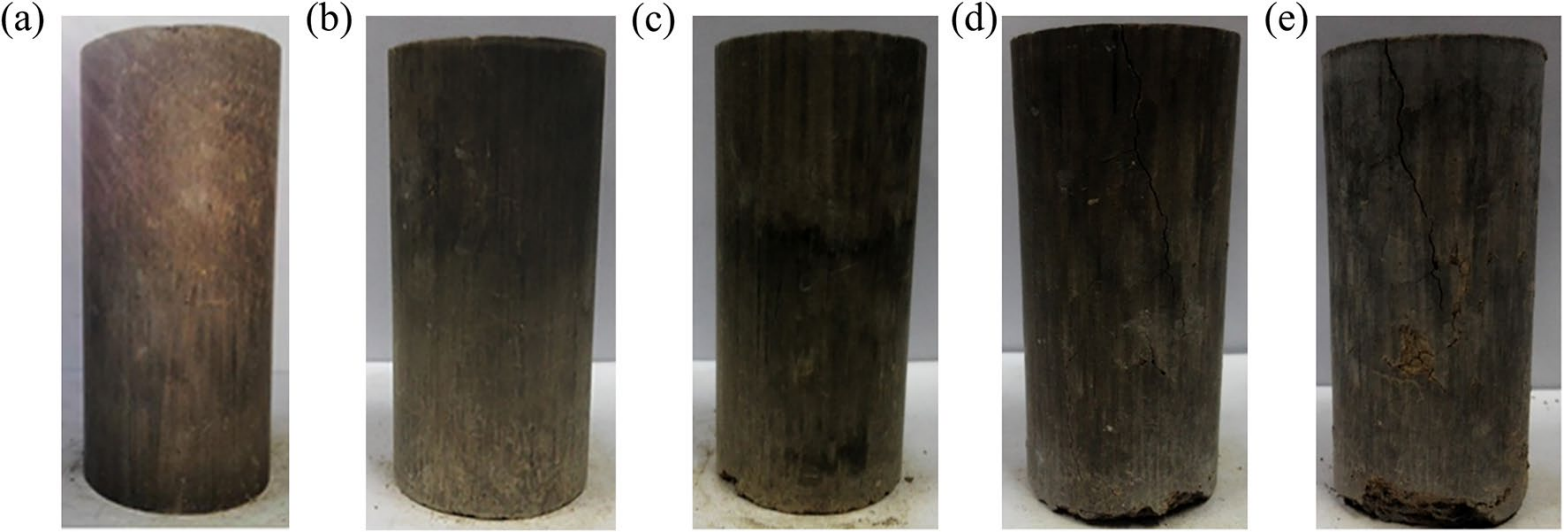
Appearance changes of cured chromium contaminated soil samples with dry and wet cycles at (**a**) 0 times; (**b**) 2 times; (**c**) 4 times; (**d**) 8 times; and (**e**) 16 times.

**Figure 5:**
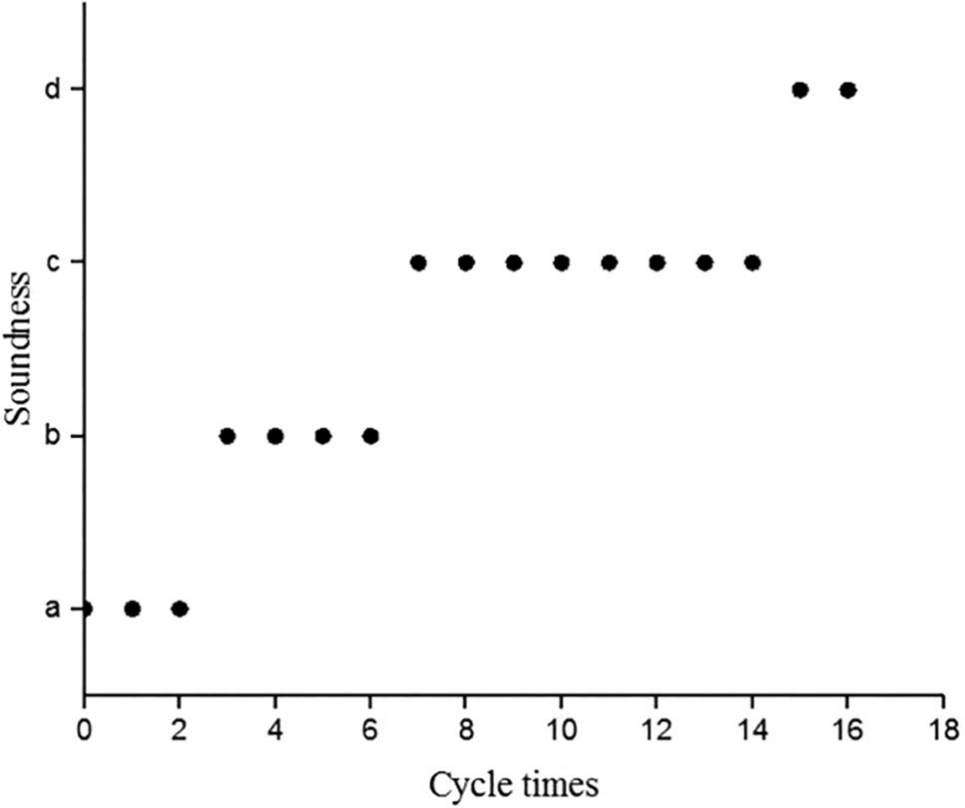
Soundness evaluation results of cured chromium contaminated soil samples.

Figure 4 is a photograph of the appearance change of a solidified 5000 mg/kg chromium-contaminated-soil sample after a dry–wet cycle. The soundness-evaluation results of the sample after each dry–wet cycle are shown in Fig. 5.

Figures 4 and 5 show that, after two dry–wet cycles of the joint repair of chromium-contaminated soil, the appearance of the sample did not significantly change, compared with 0 cycles, the surface changed from smooth to rough. Slight cracks appeared from the fourth cycle. Obvious cracks appeared in the sample at the end of the eighth cycle, and a small part of the sample fell off. The sample began to show obvious cracks from the end of the 15th dry–wet cycle, and large pieces of slack simultaneously appeared. The sample was subjected to 16 wet and dry cycles, and soundness was still not at e–h level, indicating that the joint repair of chromium-contaminated soil had strong resistance to dry and wet cycles.

### Combined repair of chromium-contaminated-soil microstructure changes under action of dry–wet cycles

**Figure 6:**
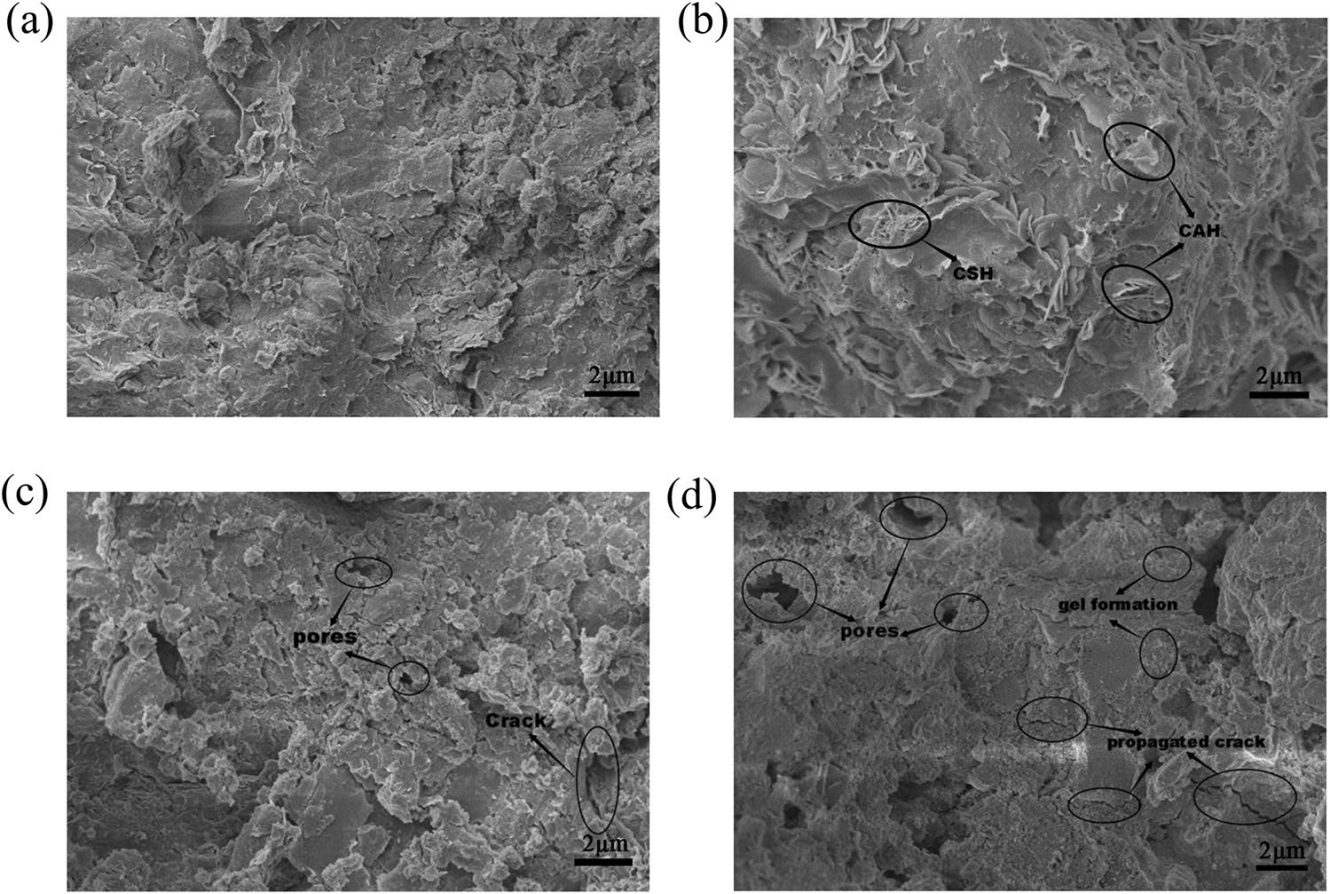
SEM images of 5000 mg/kg chromium contaminated soil specimens after different dry wet cycles at (**a**) 0 times; (**b**) 2 times; (**c**) 8 times; and (**d**) 16 times.

**Figure 7:**
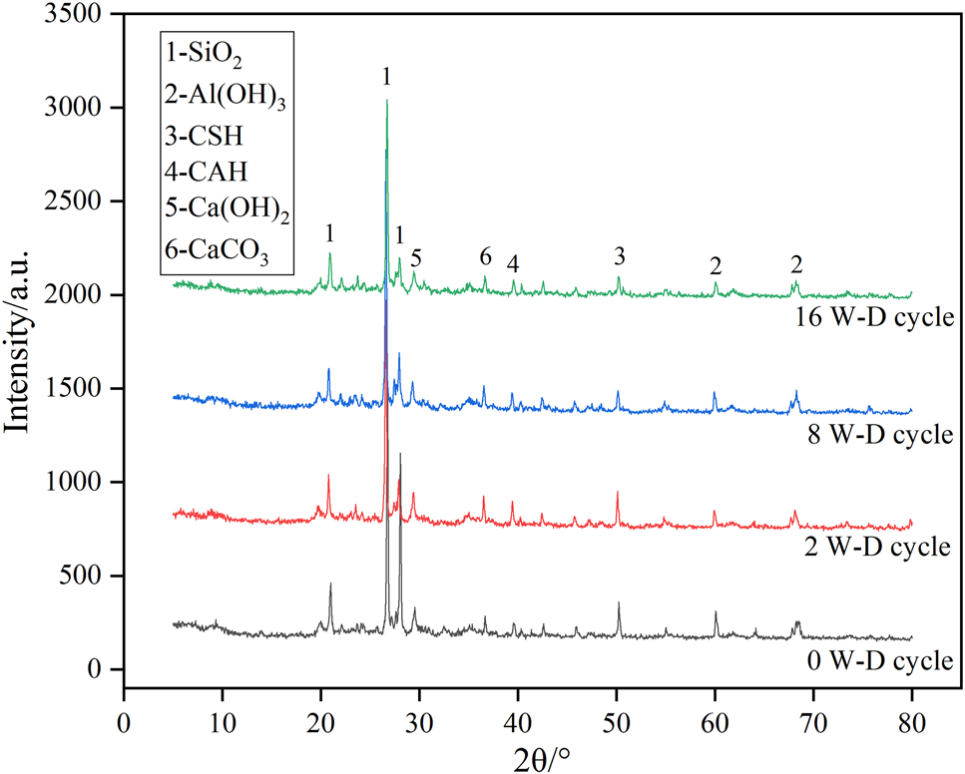
XRD pattern of 5000 mg/kg chromium contaminated soil specimen after different dry wet cycles.

After the joint repair of chromium-contaminated-soil specimens underwent a certain number of wet and dry cycles, the strength, leaching characteristics, and appearance of the specimens significantly changed. From the microstructure, there had to be corresponding changes. Therefore, scanning electron microscope (SEM) and X-ray diffraction (XRD) were used to further analyze the microstructure changes of specimens with different chromium content levels under the action of different wet and dry cycles, as shown in Figs. 6 and 7.

Figure 6 shows that the combined repair of chromium-contaminated soil after 28 days of curing had many pores in the specimen at 0 dry–wet cycles (standard sample), the physical and chemical interaction between the cement hydrate and the soil in the repair preparation still continued, and there were platelike calcium hydroxide crystals on the surface. After two dry–wet cycles, the contaminated soil was denser, and the overall structure was more complete than that in the samples without dry–wet cycles. The plate-shaped calcium hydroxide crystals were reduced, and a large number of fibrous and flocculent hydrated gels could be seen on the surface of the structure. This shows that the reaction between remediation agent and chromium-contaminated soil continued, which is consistent with the law that strength did not drop but rose during the two dry and wet cycles in the unconfined-compressive-strength test. After the test piece had undergone 8 dry–wet cycles, the surface of the test piece not only had a large increase in pores, but also had local cracks, indicating that the structure of the test piece was damaged under the action of the dry–wet cycle, which is consistent with the unconfined compressive strength found in the experiment, coinciding with a sharp drop. After 16 wet and dry cycles, the surface of the specimen not only showed a large number of pores and cracks, but also had obvious roughness. It showed that the dry–wet cycle effect caused the hydration products and cement materials in the soil to be destroyed and dissolved out, and the coupling and supporting forces between soil particles are weakened, and the strength of the soil is reduced accordingly, which was consistent with the macroscopic test results.

Figure 7 shows that the main crystal phases of the chromium-contaminated soil were SiO_2_ and Al_2_O_3_ for the samples that did not undergo a dry–wet cycle. A small number of CSH, CAH, Ca(OH)_2_, and CaCO_3_ crystals could also be detected from the diffraction peaks. Cr^3+^ and Cr^6+^ formed hydroxide precipitates in a highly alkaline environment and wrapped them on the surface of cement, hindering their contact reaction with water. Compared with 0 cycles, SiO_2_ and Al_2_O_3_ in the second cycle were decreased, while the contents of CSH, CAH, Ca(OH)_2_, and CaCO_3_ significantly increased. This is because in the process of dry and wet cycles, the sample is fully exposed to moisture and air, so the hydration, depolymerization-cementation, pozzolanic, and carbonation reactions between composite preparation and chromium-contaminated soil continued. After two dry–wet cycles, more hydration products were generated than in the specimens without dry–wet cycles, which filled the pores between the particles of the solidified body, effectively blocking the permeability of the pores, and making the contaminated soil denser, and more structured and complete. At the same time, the full progress of the hydration reaction also delayed the damage rate of the water body to the soil in the dry–wet cycle, so that the soil could maintain a certain strength in the harsh environment, which is consistent with the above-mentioned growth trend of the soil strength. At the same time, the extension of a large amount of fibrous calcium silicate hydrate greatly increased the internal specific surface area of the soil. Free-state Cr^3+^ and Cr^6+^ were adsorbed or produced hydroxide precipitation and filled in the pores of the soil, and free ion concentration was also greatly reduced, which is consistent with the above ion-leaching test results. For the specimens with 8 dry and wet cycles, the content of hydration products such as CAH and CSH was reduced. This is due to a series of evolutionary processes such as the expansion of local microcracks, the increase in macropores, the appearance of internal cracks in the contaminated soil, and the appearance of cracks and peeling on the outside of the contaminated soil. Structural integrity was destroyed, and strength was accordingly reduced. By 16 wet and dry cycles, a large amount of fibrous CSH disappeared, which weakened the cementation between soil particles. At this time, the heavy-metal ions originally wrapped in the contaminated soil solidified the body and the external environment, the contact area with the water was increased, the pH value of the environment was decreased, hydrate CSH was decalcified, and Ca/Si ratio was decreased. This reduced the adsorption capacity of the compound formulation to chromium ions, so that chromium ions were dissolved out of the soil.

## Conclusion

This study used calcium polysulfide/synthetic zeolite/cement to repair chromium-contaminated soil. Through the dry–wet cycle test, the durability of the chromium-contaminated soil after repair is analyzed from the perspectives of unconfined compressive strength, toxic leaching concentration, quality loss, and microscopic characterization. From the test results, we can draw the following conclusions:

1. The unconfined compressive strength of composite preparations for repairing chromium-contaminated soil first increased with the increase in the number of dry and wet cycles; when it reached the maximal value, it gradually decreased as the number of dry and wet cycles continued to increase, and the decrease rate became slower. Peak strength of the solidified soil with high content of chromium-contaminated soil appeared late, and the peak value was low. This is because the higher the chromium ion content was, the more serious the delay of the hydration reaction of the composite preparation was, and the more obvious the weakening effect on the strength of the solidified body was. The change process of the unconfined compressive strength of the chromium-contaminated soil in the dry–wet cycle is the dynamic balance between the strength increase caused by the hydration reaction of the composite preparation and the strength reduction caused by the dry–wet cycle, which led to the high repair of the composite preparation. The strength effect of soil contaminated by chromium was worsened.
2. In the initial stage of the dry–wet cycle, the concentration of Cr(VI) and total chromium in the leaching solution was decreased. After that, as the number of dry–wet cycles increased, the leaching concentration was increased. However, the changes of solidified low- and medium-content chromium-contaminated-soil bodies were relatively stable regardless of Cr(VI) or total chromium. The concentrations of Cr(VI) and total chromium in the leaching solution of solidified soils with high content of chromium-contaminated soil were both high, they increased with the increase in the number of wet and dry cycles, and the increase in total chromium was the most obvious.
3. With the increase in the number of dry and wet cycles, the cumulative mass-loss rate of the chromium-contaminated soil solidified by the compound formulation was gradually increased. The higher the chromium concentration of the contaminated soil was, the greater the cumulative mass-loss rate. However, after 16 cycles, the cumulative mass-loss rate of both was less than 1%.
4. Through scanning-electron-microscopy and XRD analyses, the microstructure changes of composite preparations to repair chromium-contaminated soil were consistent with macromechanical-strength and toxic-leaching changes. From a microscopic point of view, the engineering properties of composite preparations for repairing chromium-contaminated soils were revealed.

## Materials and methods

### Preparation of chromium-contaminated soil

The test soil samples were taken from uncontaminated clean soil 2 km away from a chromium slag storage site in Shenyang, China. The physical and mechanical properties, and chemical-composition determination results of the soil samples are shown in Tables 3 and 4. Before the experiment, the soil was dried to a constant weight at 60 °C and crushed through a 2 mm sieve for use. Considering that the concentration of pollutants in natural soil is not uniform, and the heterogeneity of the soil complicates mixture analysis, to facilitate comparison, we simulated the actual situation of contaminated soil near the Shenyang chromium slag storage yard, artificially prepared Cr(VI) and Cr(III)-containing chromium-contaminated soil, and carried out research on the remediation technology of chromium-contaminated soil. In view of the high solubility of NO_3_^−^ and K^+^, and little interference with the curing-reaction process^25^, we used K_2_CrO_4_, Cr(NO_3_)_3_ (analytical pure grade) reagents to prepare a solution of a certain mass concentration, and mixed it with a spare soil sample to produce Cr(VI) content of 1000, 3000, and 5000 mg/kg, respectively. Contaminated soils with different chromium content had Cr(III) content that was 7/6 times the Cr(VI) content.

**Table 3:** Basic physical and mechanical properties of soil.

Index	Moisture content/%	Soil natural density/(g/cm^3^)	Specific gravity	Saturability/%	Particle composition (% by weight)	
					Sand grains	Clay particle
Numerical value	21.0	1.99	2.71	91.3	42	32

Index	Optimum water content/%	Maximum dry density/(g/cm^3^)	Liquid limit/%	Plastic limit/%	Plastic limit index	Void ratio
Numerical value	22.0	1.72	30.6	18.7	11.9	0.657

**Table 4:** Soil chemical composition.

Items	pH	Organic matter/g/kg	CEC/cmol/kg	Al_2_O_3_/%	Fe_2_O_3_/%	MgO/%	CaO/%	Na_2_O/%	K_2_O/%	P_2_O_5_/%	SiO_2_/%
Silty clay	7.9	65.6	20.9	31.1	1.7	1.0	1.6	3.7	1.2	0.4	53.2

### Repair material

The main component of reducing agent CaS_x_ used in the experiment was polysulfide (S_x_^2−^, x = 2–6, mainly 5), and it was purchased from China Lianyungang Lanxing Industrial Technology Co., Ltd. with a volume fraction of 29%. The chemical-reaction equation is

1 $$ {\text{Cr}}_{{2}} {\text{O}}_{{7}}^{{{2}{-}}} + {\text{3CaS}}_{{5}} + {\text{8H}}^{ + } = {\text{2Cr}}\left( {{\text{OH}}} \right)_{{3}} \left( {\text{s}} \right) + {\text{15S}}\left( {\text{s}} \right) + {\text{3Ca}}^{{{2} + }} + {\text{ H}}_{{2}} {\text{O}} $$

The curing-agent cement used in the test was P.O42.5 ordinary Portland cement (OPC), and its chemical composition is shown in Table 5.

**Table 5:** Chemical composition of synthetic zeolite and cement.

Chemical composition	SiO_2_	A1_2_O_3_	CaO	MgO	Fe_2_O_3_	K_2_O	Na_2_O	TiO_2_	SO_3_	Loss on ignition
Cement/%	21.87	5.69	62.25	3.12	3.80	1.41	0.35	–	1.02	0.48
Synthetic zeolite/%	50.29	14.17	3.42	1.96	7.23	1.05	20.88	0.55	–	–

**Figure 8:**
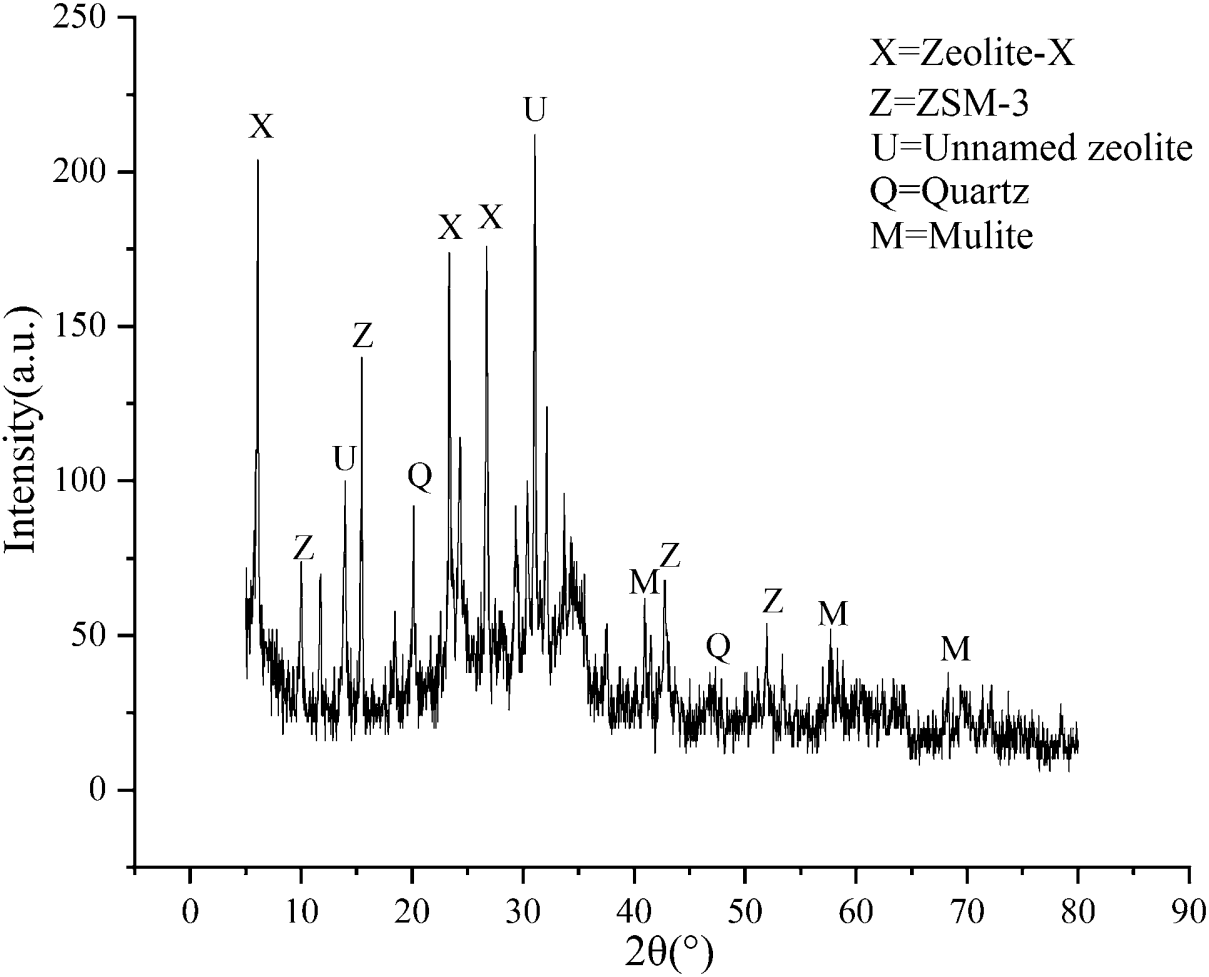
XRD pattern of zeolite synthesized from fly ash.

The absorbent fly-ash synthetic zeolite used in the experiment was prepared with the alkali fusion-hydrothermal method: The fly ash and NaOH were uniformly mixed according to a mass ratio of 1:1, and the mixture was then placed into a crucible, and then into a muffle furnace with a controlled temperature of 500 °C for roasting. After 3 h, it was taken out and ground into powder. To obtain alkali-melted fly-ash clinker, we placed the fly-ash clinker into a beaker, added distilled water at a solid-to-liquid ratio of 15/100, placed it on a magnetic stirrer, stirred at a constant speed of 200 r/min at 90 °C for 2 h, took it out, put it into a hydrothermal reactor, and placed it in an oven to crystallize at 105 °C for 9 h. We then washed it with deionized water and 95% ethanol to a pH of 7–9, dried, and ground it to obtain fly-ash synthetic zeolite powder. The chemical composition is shown in Table 5, and XRD is shown in Fig. 8.

The synthetic zeolite crystal phase prepared by alkali fusion–hydrothermal method was more complicated. X-type zeolite phase (K_86.5_Al_86.5_Si_105.5_O_384_) appeared at 2θ of 7.1, 23.2, and 27.4, and the ZSM-3 type zeolite phase (Na_1.88_Al_2_Si_2.77_O_9.48_) appeared at 2θ of 10.1, 15.8, and 43.2. An unnamed zeolite phase (Na_1.96_Al_2_Si_6.84_O_17.66_) also appeared. It is speculated that the aluminosilicate component in fly ash was effectively reorganized, which may be because fly ash contains silica and alumina necessary for the formation of zeolite, resulting in the alkali fusion–hydrothermal process. The pulverized coal ash was dissolved by hydroxyl ions at high temperatures, condensed to form aluminosilicate gel, which was then crystallized with the help of Na^+^ to form different zeolite structures.

## Experimental methods

1. Specimen Preparation and Maintenance
  The solidified chromium-contaminated soil was used as pavement-base layer filler. In order to facilitate long-term safe and stable use, the unconfined compressive strength of the soil after repair had to reach 10 MPa. The leaching concentration complies with the Technical Specification for Environmental Protection of Chromium Slag Pollution Treatment (HJ/T301-2007): hexavalent chromium leaching concentration ≤ 0.5 mg/L and total chromium leaching concentration ≤ 1.5 mg/L^26^.
  In the previous experiment, CaS_5_ was used as the reducing agent, synthetic zeolite was used as the adsorbent, and cement was used as the curing agent to conduct single-factor analysis of the joint remediation of chromium-contaminated soil. It was determined that the optimal ratio range of the solidified body for the joint repair of chromium-contaminated soil was 2–4 times of CaS_5_ content, 5–15% of synthetic zeolite, and 10–20% of cement. Taking CaS_5_, synthetic zeolite, and cement as factors, and unconfined compressive strength and toxic leaching measured by 28-day curing as indicators, an L_9_(3^4^) dual-index orthogonal test was performed. The factor levels are shown in Table 6.
  **Table 6:** Factor level table of orthogonal test.

Level	A: CaS_5_		B: synthetic zeolite		C: cement	
	Times	Dosage/mL	Ratio/%	Dosage/g	Ratio/%	Dosage/g
1	2	8.4	5	20	10	40
2	3	12.6	10	40	15	60
3	4	16.8	15	60	20	80
  In the test, we first dissolved CaS_5_ in deionized water. From Table 3, the optimal moisture content of the soil was 22%. Therefore, the quality of deionized water was maintained at 22% of the total mass of dry contaminated soil, synthetic zeolite and cement. Then mixed with chromium-contaminated soil and stirred for 30 min. After, we added fly ash synthetic zeolite, and mixed and stirred for 30 min, lastly adding cement, and mixing and stirring it for 10 min. The mixed sample was prepared with the static-pressure method, and the dry density of the sample was controlled to 95% of the maximal dry density of the corresponding mixed soil. The sample-preparation mold had an inner diameter of 5 cm and a height of 10 cm. After the sample was prepared, it was directly removed from the film, weighed, placed in a sealed plastic bag, labeled, and placed in a standard curing room (temperature 20 ± 2 °C, relative humidity above 95%) After curing for 28 days, we took it out for testing. The evaluation indicators were unconfined compressive strength and toxic leaching. Through range analysis, the optimal ratio of the composite preparation made of CaS_5_, synthetic zeolite, and cement was determined.
  The dry–wet-cycle test was completed on the basis of determining the optimal mixing ratio. The specific ratio is shown in Table 7.
  **Table 7:** Ratio of test samples.

Serial number	Cr^6+^ content/mg/kg^1^	Cr^3+^ content/mg/kg^1^	Contaminated soil/g	CaS_5_/times	Zeolite synthesized from fly ash/g	Cement/g	Deionized water/mL
1	1000	1165	260	3	60	80	88
2	3000	3500	260	3	60	80	88
3	5000	5835	260	3	60	80	88
2. Dry–Wet Cycle Test
  According to ASTM D4843-88 (reapproved 2016) and Takeshi (1993), the dry–wet cycle test was used^27,28^. The test piece was dried in an oven at 60 ± 2 °C for 24 h, and the sample was taken out of the oven, placed at room temperature of about 20 °C for 1 h, and then completely immersed in distilled water. We placed it in an incubator at 20 ± 3 °C for 23 h, and cleaned the surface of the sample that was taken out. We observed possible cracking, damage, and other phenomena, and conducted unconfined-compressive-strength, quality, and toxicity-leaching tests. A total of 16 cycles were performed.
3. Unconfined-Compressive-Strength (UCS) Measurement
  Unconfined compressive strength (UCS) could be tested according to ASTM D2166-06^29^, and the axial strain rate was 1 mm/min.
4. Determination of Leaching Concentration
  Considering that the solubility of oxygen-containing anions, such as hexavalent chromium, reaches the maximum at neutral to weakly alkaline pH values^30,31^, the leaching experiment adopted China's HJ/T 557—2010 solid-waste-leaching toxicity-leaching method–horizontal-oscillation method; the extractant was deionized water, the leaching concentration of Cr(VI) and total chromium was measured by a Z-2000 atomic absorption spectrophotometer, and each experiment was repeated 3 times.
5. Mass loss determination
  The durability of the joint repair of chromium-contaminated soil can also be evaluated by examining the mass loss of the specimen during a dry–wet cycle. The quality change is determined by weighing the mass of the test piece in each cycle. Before weighing the test piece, the surface of the test piece was lightly brushed with a brush. The percentage of mass loss was based on the first dry-mass measurement. After each dry–wet cycle, it was necessary to calculate cumulative mass-loss rate C (%) according to the weight change of the weighed sample according to Formula (2), and judge whether the sample was damaged from appearance. When C was greater than 30% or the sample was damaged during the test, the durability of the sample could not meet the requirements^32^.
  The formula for calculating cumulative mass-loss rate was
  2 $$C = \sum\limits_{i = 1}^{n} {\frac{{W_{i} }}{{M_{0} }}} = \sum\limits_{i = 1}^{n} {\frac{{\left( {M_{0} - M_{n} } \right)}}{{M_{0} }}} ,\quad \left( {n = 1,2,3 \ldots ,16} \right)$$
  where $W_{i}$ is the mass loss of the sample after the *i*th cycle (mass of dry matter remaining in the beaker after the *i*th cycle), $M_{0}$ is the initial dry mass of the sample (mass measured at 28 days), and $M_{n}$ is the dry mass of the sample in the *n*th cycle.

Describe the soundness of the sample after each dry and wet condition, as shown in Table 8^33^.

**Table 8:** Soundness evaluation method.

Soundness level	Appearance of crack	Appearance of breakage
a	Remain the same	
b	Microcrack appearance Partly crack appearance	Surface delamination
c	Appearance of obvious crack, partly	Appearance of exfoliation, partly
d	Appearance of obvious crack, totally	Appearance of exfoliation, totally
e	Specimen deterioration, partly or totally	
f	The whole sample appears to collapse and collapse, but the basic shape still exists	
g	Specimen deterioration,Maassive form	
h	Specimen deterioration, Grain refining	
